# Risk Evaluation of *Verticillium* Wilt on Cabbage Grown in Soil Reused from Sediment Basins

**DOI:** 10.1264/jsme2.ME25017

**Published:** 2025-09-27

**Authors:** Hiroyuki Yamada, Masahito Banba, Keisuke Hoshino, Yukari Nakatsuji, Kentaro Ikeda

**Affiliations:** 1 Gunma Agricultural Technology Center, Isesaki 379–2224, Japan; 2 Aguro-Kanesho Co., Ltd, Tokyo 100–0005, Japan; 3 Faculty of Bioscience and Applied Chemistry, Hosei University, Tokyo 184–8584, Japan

**Keywords:** sediment basin, reusing soil, soil erosion, *Verticillium* wilt, quantitative nested real-time PCR

## Abstract

There is a growing demand for the reuse of sediment basin soil in cabbage fields; however, reusing soil poses a potential challenge of spreading *Verticillium* wilt to the fields via pathogen-infested sediments. We evaluated the density of the *Verticillium* wilt pathogen in sediment basin soil using a quantitative nested real-time polymerase chain reaction assay and its incidence using pot cultivation tests. We detected low pathogenic DNA levels in the sediment, coupled with a low *Verticillium* wilt incidence and severity in the pot experiment, indicating a low risk of spreading *Verticillium* wilt with the reuse of sediment basin soil.

Tsumagoi in Gunma prefecture, one of the largest cabbage-producing regions in Japan, witnesses a high degree of soil erosion frequently in cabbage fields. This is attributed to 72% of arable land in this region having a slope ≥4° and 22% having a slope ≥8° (Kanuma, 2021). Shiono *et al.* (2002) estimated that the erosion rate of all cabbage fields in Tsumagoi ranged between 0.2 and 70.6 t ha^–1^ y^–1^ (averaging 10.5 t ha^–1^ y^–1^). The soil erosion rate in Tsumagoi cabbage fields is considered to be high because the average erosion rate is higher than the tolerance value of 5.0‍ ‍t‍ ‍ha^–1^‍ ‍y^–1^ for water erosion, as defined by the Organization for Economic Cooperation and Development (OECD) (2001). Since soil erosion removes fertile topsoil and causes environmental pollution in downstream rivers and lakes, 92 sediment basins were built in this region to capture eroded soil and prevent it from reaching downstream rivers.

The frequency of heavy rains in Japan has recently increased (Japan Meteorological Agency, 2024a). Heavy rain increases the amount of erosion and likely reduces the crop yield. Poudel *et al.* (1999) reported a 56% higher reduction in cabbage yield in the upper part of an eroded sloping field in the Philippines than in the lower part of the slope, where the eroded topsoil was deposited. Evidence indicates that some cabbage fields in Tsumagoi have lost their fertile topsoil. The soil carbon content in 14 fields in Tsumagoi decreased by 13% between 1979 and 2015, primarily because of the loss of topsoil (Kanuma *et al.*, 2022). To overcome this issue, there is a growing demand to reuse the soil accumulated in sediment basins.

However, the reuse of this soil presents some challenges. Cabbage production in this region has been affected by *Verticillium* wilt since 1994 (Shiraishi *et al.*, 2000). *Verticillium* spp. are well-known soil-borne pathogens that infect many crops and cause significant economic loss (Klosterman *et al.*, 2009). The main pathogenic species prevalent in this region include *Verticillium dahliae* and *V. longisporum* (Banno *et al.*, 2011; Ikeda *et al.*, 2012), which survive for years using microsclerotia as a resting structure in the soil (Clarkson and Whipps, 2002). *Verticillium* wilt likely spreads by natural (via soil and water) and human-mediated processes (via tractor tires and irrigation water) (García-Cabello *et al.*, 2012; Baroudy *et al.*, 2018; Ikeda and Osawa, 2020). Therefore, farmers are concerned about the risk of *Verticillium* wilt spreading to more areas through pathogen-infested sediment basin soils.

To the best of our knowledge, the occurrence of soil-borne pathogens in sediment basins has not yet been investigated. We herein exami­ned the amount of *Verticillium* wilt pathogen DNA in sediment basins using quantitative nested real-time PCR (QNRT-PCR) (Banno *et al.*, 2011). In addition, we aimed to assess the incidence and severity of *Verticillium* wilt in eggplant seedlings grown in pots containing soil collected from sediment basins in order to evaluate the risk of *Verticillium* wilt infection by reusing sediment basin soil.

Tsumagoi (36°51′17″ N, 138°53′00″ E) is located at an altitude of 700 to 1,500‍ ‍m in the central region of Honshu, the largest of Japan’s four main islands. Between 1991 and 2020, the region experienced a mean annual temperature of 7.4°C and a mean annual precipitation of 1,503‍ ‍mm (Japan Meteorological Agency, 2024b). The soil type in the region is classified as Andisol. Cabbage is cultivated on approximately 3,000 ha of the 3,390 ha of arable land in summer (May to October) (Village Office of Tsumagoi, 2021). This is one of the largest monoculture sites used for cabbage cultivation in Japan. Of the 92 sediment basins in Tsumagoi, we selected 30 sediment basins that were easily accessible by roads for sampling (Fig. 1). Soil samples were collected between late June and mid-July 2021 for QNRT-PCR. Some sediment basins were partially flooded; therefore, all samples were collected above the waterline. Soil samples were collected using a trowel from a depth of up to 20‍ ‍cm at five random points within each basin, and the five sediment samples were mixed to produce one representative sample per basin.

The quantity of *Verticillium* spp. in the sediment soil was detected using a modified version of the QNRT-PCR method described in Banno *et al.* (2011). Specifically, we performed a soil sample pretreatment to improve DNA extraction efficiency. After drying 200‍ ‍g of the collected well-mixed soil at 60°C, we pulverized it using a ball mill (Mixer Mill MM400; Retsch) (Koyama *et al.*, 2015), extracted DNA according to the method described by Sato *et al.* (2010), and purified the extracted DNA using the DNeasy Plant Mini Kit (Qiagen). First-round PCR was performed as described by Banno *et al.* (2011), with the exception that 2‍ ‍μL of the purified DNA extract was used as the template. Second-round PCR was conducted according to the QNRT-PCR protocol described in the same study. Mean cycle threshold (Ct) values were calculated from the Ct data obtained from three replicates of each soil DNA extraction.

Fig. 2 shows the Ct values of soil samples from each sediment basin. We detected *V. dahliae* DNA in soil sampled from 13 sediment basins, but not the other 17 basins. The Ct values of sediment basin soils in which *V. dahliae* DNA was detected ranged between 23.4 and 42.1. The Ct values in 11 sediment basins were >27, whereas values in sediment basins ID57 and 92 were 23.4 and 24.0, respectively. The DNA of *V. longisporum* was detected in only one of the 30 sediment basins, with a Ct value >40, indicating that the DNA amount was low in the basin.

This is the first study to report the detection of *Verticillium* wilt pathogen DNA in the soil of a sediment basin. Of the 30 sediment basins sampled, we detected *V. dahliae* DNA in 13 and *V. longisporum* DNA in one (Fig. 2). These results suggest that sediment basins serve as potential reservoirs for soil-borne pathogens, which was previously unrecognized. Ikeda *et al.* (2012) reported the spatial distributions of *V. dahliae* and *V. longisporum* in the soil of Chinese cabbage fields, with *V. dahliae* being the most common species in the Tsumagoi region. This is consistent with the present results because *V. dahliae* was detected more frequently than *V. longisporum* in the sediment basins.

However, of the 13 sediment basins in which *V. dahliae* DNA was detected, 11 had Ct values >27. The higher the Ct value, the lower the amount of pathogen DNA detected. According to the study by Banno *et al.* (2011), Ct values in cabbage fields in Tsumagoi Village ranged between approximately 21 and at least 35 in severely infested fields and those where the pathogen was not detected. Moreover, a correlation was observed between disease incidence and Ct values and also between Ct values and disease severity in resistant and susceptible cabbage cultivars. For example, even the cabbage cultivar that was the most susceptible to *Verticillium* wilt, ‘YR Shinpu’ (Gunma Agricultural Technology Center, 2016), was free from severe symptoms at Ct values >27. In the moderately resistant cabbage cultivar ‘Teruyoshi’, symptoms begin to appear at Ct values <25, while in the highly resistant cultivar ‘YR Ranpo’, severe symptoms only developed at Ct values <22. Therefore, when the Ct value is <22, even highly resistant cultivars are at an increased risk of developing *Verticillium* wilt. Based on these results, if soils from sediment basins ID57 and 92 (with Ct values of 23.4 and 24.0, respectively) is reused, the cultivation of resistant cabbage varieties, such as ‘YR Ranpo’, is recommended. In contrast, soils from the other 11 sediment basins had a low risk of disease. However, the physicochemical properties of the soils used in the present study differed from those investigated by Banno *et al.* (2011), which may have affected disease susceptibility. In addition, we observed slight differences in the pretreatment methods applied to the soil samples used for the QNRT-PCR ana­lysis. Therefore, we performed a bioassay using sediment basin soils for a further disease risk evaluation.

Small-scale experiments were conducted to assess the incidence and severity of *Verticillium* wilt. Based on the Ct values obtained from the initial 30 sediment basins, we selected seven basins to cover a wide range of Ct values (indicated by black dots in Fig. 1) and collected soil samples from these sites on September 13, 2021 using the above-described method. We mainly used these soils for a preliminary bioassay, aiming to evaluate the validity of the detection methods before conducting the main experiment. In addition, for the main bioassay, we collected soil samples from the same seven sediment basins again on May 25, 2022. In the preliminary experiment, we mixed the sediment basin soil with commercially available potting soil (“Baiyodo”, Setogaharakaen) in a 3:7 (v/v) ratio. In the main experiment, soil samples from each sediment basin were thoroughly mixed and added to polyvinyl chloride pots (9‍ ‍cm in diameter, 8‍ ‍cm in height). As the positive control, we used sterile soil-wheat bran medium (soil: wheat bran: distilled water=4:1:1 [v/v/v]) (Ikeda *et al.*, 2015). This medium was inoculated with the *V. dahliae* EV-KY2 strain, cultured for one month, and mixed with commercially available potting soil (“Baiyodo”, Setogaharakaen) at 0.3% (w/w). The sediment basin soils collected for the main experiment, as well as the soils used as the positive and negative controls, were subjected to a QNRT-PCR ana­lysis. The Ct value for positive control soil used in the main test was 10.4. The positive control was not intended for direct comparisons with sediment basin soils, but rather to confirm that the eggplants we used in the experiment appropriately responded to the pathogen and also that the experimental system was functioning properly under conditions favorable for disease development. The same potting soil without the pathogen was used as the negative control. In the preliminary and main experiments, we prepared three and five pots for each sediment basin, and transplanted three eggplant seedlings (“Senryo-nigou” variety; Takii) into each pot on June 2 and August 24, 2022, respectively. Eggplants were selected for this experiment because of their high susceptibility to *V. dahliae* (Hagiwara, 1990). Furthermore, we selected *V. dahliae* for this experiment because it is more common in this region than *V. longisporum* (Ikeda *et al.*, 2012). We cut the stems near the plant base and main roots using a cutter and exami­ned the degree of browning in cross-sections in the preliminary and main experiments on July 7 and September 27, 2022, respectively. Based on these observations, the disease incidence and severity of *Verticillium* wilt were calculated using the following formulas:

Disease incidence (%)=100×(Number of symptomatic plants/Total number of plants surveyed) (1)

Disease severity=100×Σ (Number of diseased plants per severity category×Severity index)/(4×Total number of plants surveyed) (2)

In addition, for plants that developed symptoms during the bioassay, pathogens were isolated from the diseased tissues and identified.

Table 1 shows *Verticillium* wilt incidence and severity in eggplant seedlings grown in the sediment basin soil. It also shows the Ct values for *V. dahliae* obtained from soil samples collected in June–July 2021 and May 2022 as well as the pathogen isolation results from diseased plant tissues.

ID57 served as an important case supporting the validity of the combined evaluation method using both QNRT-PCR and the bioassay. In the preliminary experiment, ID57 exhibited a disease incidence of 22.2% and a severity score of 8.3. Moreover, we achieved successful *V. dahliae* re-isolation from diseased tissues. Although we observed no disease symptoms in the main experiment, the Ct values of the soil collected in 2021 and 2022 were low (23.4 and 20.9, respectively), suggesting a high pathogen density. Although we did not measure Ct values for the soil used in the preliminary experiment (collected in September 2021), we confirmed disease symptoms and successful *V. dahliae* re-isolation, obtaining consistent results between disease incidence, presence of the pathogen, and re-isolation.

In contrast, several examples displayed inconsistencies between Ct values and bioassay results. For example, although the ID50 soil sample retained a high Ct value of 34.8 in 2021 (indicating a low pathogen density), the Ct value was markedly lower (*i.e.*, 18.2) in 2022, confirming pathogen density variations among the samples. However, we observed no disease symptoms in the preliminary or main experiments. Similarly, ID92 yielded low Ct values (24.0–26.8), indicating high pathogen density with no disease symptoms. Therefore, a low Ct value does not necessarily indicate disease development in the bioassay. Possible factors contributing to disease development include environmental conditions affecting eggplant susceptibility, such as soil moisture content (Emechebe, 1980) and pH (Dutta, 1981).

In contrast, despite high Ct values in ID13 and ID45 (ND and 28.7–32.6, respectively), we registered a disease incidence of 6.7% and severity score of 1.7. However, we were unable to re-isolate *V. dahliae* from diseased plants in these cases. Re-isolation involves direct pathogen isolation from plant tissues. At low disease severity levels, the amount of the pathogen within the plant may be too small for successful re-isolation.

Therefore, while we detected a correlation between Ct values and disease incidence, we were unable to achieve complete consistency across all cases. Nevertheless, except for specific cases (*e.g.*, ID57), most sediment basin soils were considered to have a low disease risk based on the combined Ct value ana­lysis and bioassay results. Previous studies reported that the risk of *Verticillium* wilt increased at Ct values <27, and even resistant cabbage cultivars developed disease symptoms at Ct values <22 (Banno *et al.*, 2011). In the present study, the Ct values in ID57 corresponded to this risk threshold, suggesting that Ct-based screening is efficient for evaluating sediment basin soil reuse potentials. Furthermore, since resistant cabbage cultivars are widely adopted in the Tsumagoi region, the actual risk of disease may remain low in the field, even when reusing soils with low Ct values.

Previous studies reported that *V. dahliae* was sensitive to anaerobic conditions and also that temperature played an important role in its survival under these conditions (Ebihara and Uematsu, 2014). For example, though *V. dahliae* is quickly killed at high temperatures (20–35°C) (Hashimoto, 1989), it survives longer at lower temperatures (<15°C) (Pullman and DeVay, 1982). In the study region, heavy rainfall events (>20‍ ‍mm of precipitation h^–1^) are concentrated between July and September, resulting in large volumes of soil and water flowing into the sediment basins. Due to their pond-like structures (Fig. 1), these basins generally retain water, causing the accumulated soil to become anaerobic. Additionally, the average daily temperature between July and September is typically higher than 15°C, which may create an unfavorable environment for *Verticillium* wilt. There is currently no information on whether soil-borne plant pathogens are present in the soil of sediment basins. Therefore, this study contributes to the expansion of knowledge on soil-borne plant pathogenic risks in sediment basins. Furthermore, sediment reuse is an important countermeasure against soil erosion and extends the lifespan of sediment basins. This study promotes the reuse of sediment basin soil in cabbage fields in Tsumagoi, which may help prevent cabbage yield loss.

## Citation

Yamada, H., Banba, M., Hoshino, K., Nakatsuji, Y., and Ikeda, K. (2025) Risk Evaluation of *Verticillium* Wilt on Cabbage Grown in Soil Reused from Sediment Basins. *Microbes Environ ***40**: ME25017.

https://doi.org/10.1264/jsme2.ME25017

## Figures and Tables

**Fig. 1.**
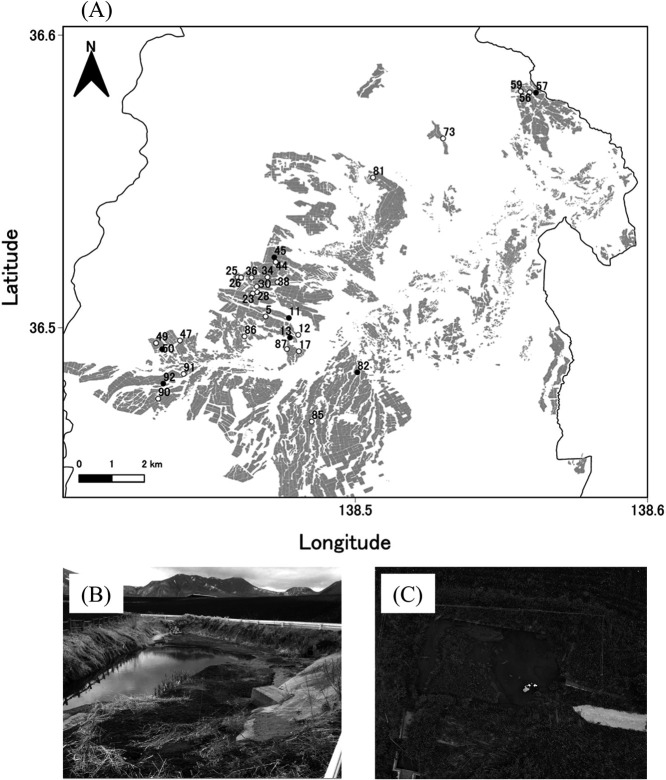
Locations of sediment basins and appearance of a typical sediment basin in the Tsumagoi region. (A) Map of sediment basins in the region. Numbers indicate sediment basin IDs. Dots indicate the locations of the 30 sediment basins sampled and the black dots indicate the locations of the seven sediment basins that provided the soil for the pot experiment. The map was created by processing field polygon data obtained from the Ministry of Agriculture, Forestry, and Fisheries. (B) and (C) show the appearance of sediment basin ID85 and 90, respectively.

**Fig. 2.**
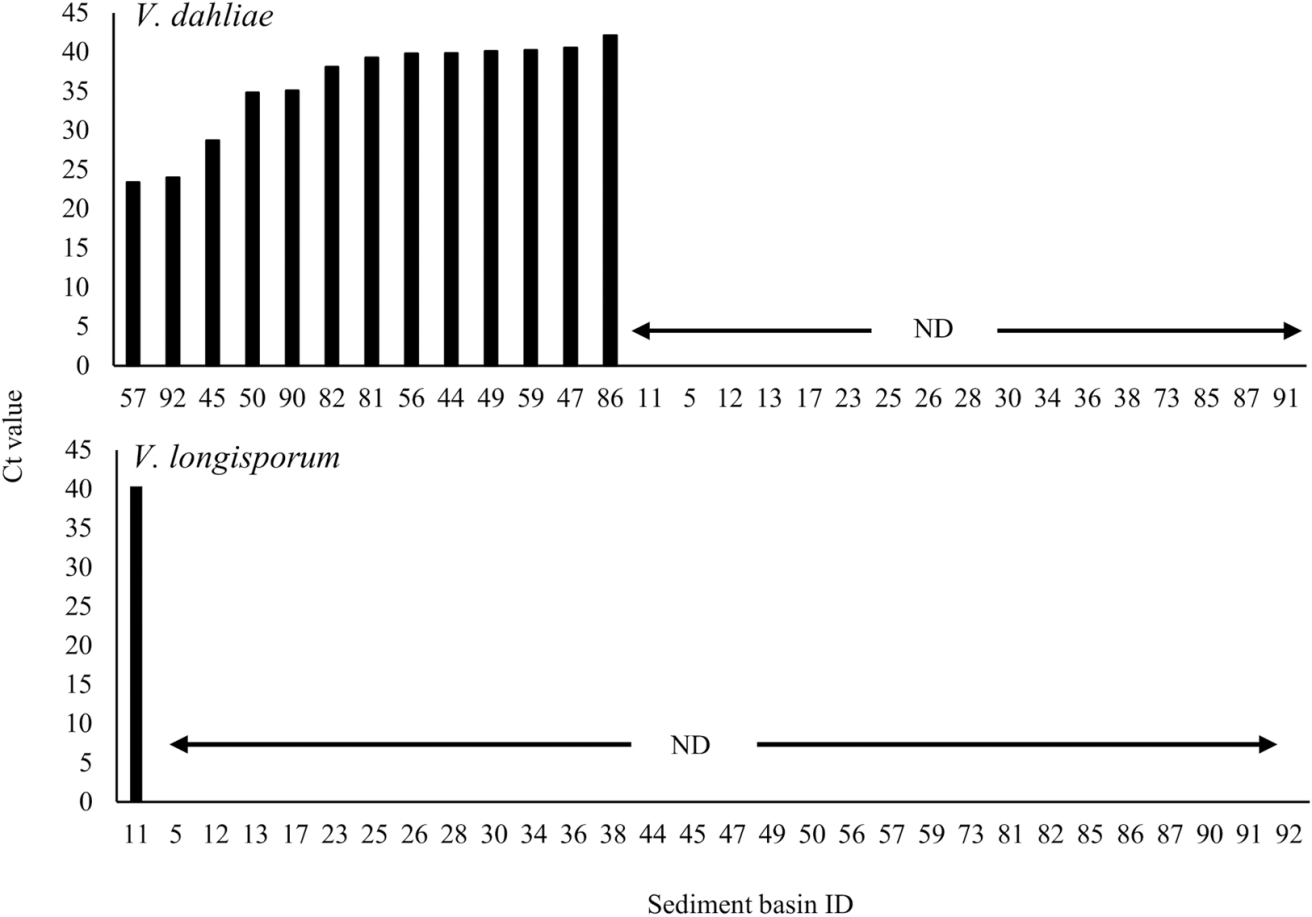
Ct values of sediment basin soils sampled in Tsumagoi, Japan. The higher the Ct value, the lower the amount of pathogen DNA detected. ND indicates that no soil-borne pathogen DNA was detected.

**Table 1.** Results of the bioassay on eggplant and associated Ct values.

Pot ID	Bioassay on Eggplant						QNRT-PCR	
	Preliminary test			Main Test				
	Incidence (%)	Severity		Incidence (%)	Severity		Ct value	
Ctrl(+)	9/9 (100)*	66.7		15/15 (100)*	60.0		—	10.4
Ctrl(–)	0/9 (0.0)	0.0		0/15 (0.0)	0.0		—	ND
13	0/9 (0.0)	0.0		1/15 (6.7)	1.7		ND	ND
45	0/9 (0.0)	0.0		1/15 (6.7)	1.7		28.7	32.6
50	0/9 (0.0)	0.0		0/15 (0.0)	0.0		34.8	18.2
57	2/9 (22.2)*	8.3		0/15 (0.0)	0.0		23.4	20.9
73	0/9 (0.0)	0.0		0/15 (0.0)	0.0		ND	ND
82	0/9 (0.0)	0.0		0/15 (0.0)	0.0		38.1	26.2
92	0/9 (0.0)	0.0		0/15 (0.0)	0.0		24	26.8
Date of tested soil sampling	September 13, 2021			May 25, 2022			Late June– mid July, 2021	May 25, 2022

The pot ID number corresponds to the sediment basin ID number shown in Fig. 2, indicating the sediment basin soil used in the pot experiment. Incidence: No. disease plants No. surveyed plants^–1^. The numbers in parentheses are percentages. Severity was calculated using the following index: Severity index 0: No symptoms, 1: Less than 25% of the vascular cross-section browned, 2: 25 to 50% of the vascular cross-section browned, 3: 50 to 75% of the vascular cross-section browned, and 4: more than 75% of the vascular cross-section browned. The Ct value indicates the value for *Verticillium dahliae*. A superscript asterisk next to the disease incidence value indicates successful *V. dahliae* re-isolation from symptomatic tissue.
